# Does temporal discounting explain unhealthy behavior? A systematic review and reinforcement learning perspective

**DOI:** 10.3389/fnbeh.2014.00076

**Published:** 2014-03-12

**Authors:** Giles W. Story, Ivo Vlaev, Ben Seymour, Ara Darzi, Raymond J. Dolan

**Affiliations:** ^1^Department of Surgery and Cancer, Centre for Health Policy, Institute of Global Health Innovation, Imperial College LondonLondon, UK; ^2^Wellcome Trust Centre for Neuroimaging, Institute of Neurology, University College LondonLondon, UK; ^3^Center for Information and Neural Networks, National Institute for Information and Communications TechnologyTokyo, Japan

**Keywords:** discounting, health, addiction, model-based, model-free, habit, preference reversal, hyperbolic

## Abstract

The tendency to make unhealthy choices is hypothesized to be related to an individual's temporal discount rate, the theoretical rate at which they devalue delayed rewards. Furthermore, a particular form of temporal discounting, hyperbolic discounting, has been proposed to explain why unhealthy behavior can occur despite healthy intentions. We examine these two hypotheses in turn. We first systematically review studies which investigate whether discount rates can predict unhealthy behavior. These studies reveal that high discount rates for money (and in some instances food or drug rewards) are associated with several unhealthy behaviors and markers of health status, establishing discounting as a promising predictive measure. We secondly examine whether intention-incongruent unhealthy actions are consistent with hyperbolic discounting. We conclude that intention-incongruent actions are often triggered by environmental cues or changes in motivational state, whose effects are not parameterized by hyperbolic discounting. We propose a framework for understanding these state-based effects in terms of the interplay of two distinct reinforcement learning mechanisms: a “model-based” (or goal-directed) system and a “model-free” (or habitual) system. Under this framework, while discounting of delayed health may contribute to the initiation of unhealthy behavior, with repetition, many unhealthy behaviors become habitual; if health goals then change, habitual behavior can still arise in response to environmental cues. We propose that the burgeoning development of computational models of these processes will permit further identification of health decision-making phenotypes.

## Introduction

Unhealthy behaviors, such as tobacco smoking, excessive alcohol intake, physical inactivity and substance misuse, account for significant morbidity and mortality worldwide (Smith et al., 2012). For example tobacco smoking was estimated to be responsible for a fifth of all deaths in the US in 2005 (Danaei et al., 2009). In the developed world these behavioral risk factors can be viewed as a direct expression of personal choice, and research has therefore been directed toward establishing their psychological determinants (Conner and Norman, 2005). Unhealthy behavior frequently has a delayed effect on health, leading researchers to hypothesize that an individual's tendency to make unhealthy choices is related to their temporal discount rate, the theoretical rate at which they devalue delayed outcomes (Grossman, 1973; Bickel et al., 2012). In recent years a large number of studies have addressed this hypothesis, forming part of a growing endeavor to identify decision-making phenotypes which correlate with maladaptive behavior (Montague et al., 2012).

Given a choice, people tend to prefer immediate rewards to those available after a delay, for example preferring to receive $10 today over $15 in a month, implying that they *discount* the value of delayed rewards relative to more immediate ones. A function which describes the pattern of discounting can be estimated by observing choices between delayed outcomes. Economic theories of rational behavior posit that goods ought to be discounted exponentially with delay (Samuelson, 1937). Formally, an outcome which has utility A if received immediately (*t* = 0) is worth *A*· δ^*t*^ if delayed *t* periods into the future. The present-time value, *V*, of receiving *A* at time *t* is thus given by:

(1) $$V(A,t)=\;A\cdot \delta^{t}$$

Here the discount rate, δ, represents the constant proportional decrease in value with each added time period of delay. However both humans and animals violate the exponential assumption of a constant proportional discount factor per unit time, appearing rather to discount rewards occurring in the immediate future more steeply than those in the distant future. In other words, delaying an immediate reward 1 day into the future decreases its implied value proportionally more than does delaying an already distant reward by 1 day (Strotz, 1957; Chung and Herrnstein, 1967; Ainslie, 1974, 1975; Mazur, 1987; Benzion et al., 1989; Green et al., 1994). The discount function estimated from observed choices is better accounted for by a hyperbolic function, written in its simplest form as follows, where *k* represents the hyperbolic discount rate:

(2) $$V(A,t)=\;A\cdot \frac{1}{\text{1}+k\cdot t}$$

A proposed alternative is the “quasi-hyperbolic” approximation to hyperbolic discounting (Phelps and Pollak, 1968; Laibson, 1997; Angeletos et al., 2001; McClure et al., 2004, 2007), which is formalized as exponential discounting, with an additional preference for immediate rewards, expressed as a second discount factor, β, applied to all time-periods except the first:

(3) $$V(A,t)=\{\begin{array}{l} A\;if\;t=0\\ A\cdot \beta \delta^{t}\;if\;t>0 \end{array}$$

The hyperbolic and quasi-hyperbolic forms qualitatively capture the observation that individuals make far-sighted plans when outcomes are distant, but reverse their choices in favor of short-term rewards when the future is reached (see Kalenscher and Pennartz, 2008 for a review). Hyperbolic or quasi-hyperbolic discounting therefore putatively imparts a psychological explanation for why people intend to perform actions they subsequently fail to carry out. In economics this is referred to as myopic preference reversal (Kirby and Herrnstein, 1995), and bears considerable relevance to health choice, where there is a marked discrepancy between health intentions and health behavior (Conner and Norman, 2005).

This article examines the relationship between temporal discounting and health behavior at two levels of analysis: *empirical* and *mechanistic* (Rescigno et al., 1987). A distinction is often made in psychology and neuroscience between empirical and mechanistic classes of model. Empirical, high-level, or descriptive models seek to capture structure in observed data, allowing predictions to be made, but lack psychological content (Lewandowsky and Farrell, 2011). Thus temporal discounting might predict markers of health behavior, without being upheld as a psychological process underlying unhealthy choice (see Frederick et al., 2002 for an historical perspective on discounting as a descriptive model). For example, discounting measures might simply be correlated with environmental factors influencing health behavior, such as peer influence. By contrast, mechanistic, low-level or explanatory models specify psychological processes at an abstract level, or seek to identify how neural systems solve computational problems (Farrell and Lewandowsky, 2010; Lewandowsky and Farrell, 2011; Montague et al., 2012). Some authors propose that *hyperbolic* discounting is a mechanistic model, i.e., a fundamental principle for choosing between delayed outcomes (for example Ainslie, 2001). This notion leads to the hypothesis that unhealthy choices directly result from hyperbolic discounting of their distant health consequences. The latter is consistent with the recent proposal that discounting is a trans-disease process underlying several impulsive pathologies (Bickel et al., 2012). To address the first, empirical, hypothesis we perform systematic review of studies which examine relationships between temporal discount rates and health behavior or health status. To address the plausibility of the second, mechanistic, hypothesis we examine whether both health behavior in the field and laboratory choice behavior fulfill the predictions of hyperbolic discounting. Predicting unhealthy choice has potential utility in guiding health behavior change interventions (Conner and Norman, 2005), while understanding the processes involved in unhealthy choice may assist in designing novel interventions.

## Can measured discount rates predict health behavior?

### Differences between discounting for health and money

Measured discount rates are not equal for all commodities, for example, people discount primary reinforcers such as food and water more steeply than money (Odum, 2011). This raises an important question: which outcome modality is the best candidate predictor of health behavior? With regard to real-world health choices the relevant delayed outcome is a health state. Indeed numerous studies have attempted to measure discount rates for hypothetical health states (Asenso-Boadi et al., 2008). Many such studies require participants to imagine a hypothetical illness, usually described in generic terms, and to trade-off the severity or duration of the illness against when they would prefer the illness to occur (for example Van Der Pol and Cairns, 2001). Individuals who are willing to accept a more severe illness occurring after a delay over a less severe immediate illness are said to discount future illness. Alternatively, the health state may be described as an improvement in health from a baseline of illness (for example Ganiats et al., 2000), where individuals who prefer immediate over delayed health improvement, and are willing to accept a small improvement in health occurring sooner, over a larger improvement at a delay are said to discount future health.

Studies of hypothetical health discounting, for both health improvements and illness, demonstrate that some important properties of monetary discounting are conserved in the health domain. Several studies find that many people indeed discount future health improvements in the conventional sense, also referred to as positive time preference for health (Lipscomb, 1989; Olsen, 1993; Bleichrodt and Johannesson, 2001). Also consistent with monetary discounting there is robust evidence for decreasing health discount rates over increasing delay (Bleichrodt and Johannesson, 2001). Furthermore, Van Der Pol and Cairns (2002) asked participants to make choices relating to a generic illness, which could be delayed into the future by means of an imaginary treatment, showing that hyperbolic discount functions fitted choices better than exponential ones (see also Van Der Pol and Cairns, 2011).

Problematically however temporal discounting for health, unlike monetary, outcomes is far from universal. A proportion of people in fact prefer to advance the timing of illness (Cairns, 1992; Redelmeier and Heller, 1993; Chapman, 1996; Chapman and Coups, 1999; Van Der Pol and Cairns, 2000, 2002, 2011; Chapman et al., 2001) or to delay health improvements (Olsen, 1993; Chapman and Elstein, 1995; Dolan and Gudex, 1995; Chapman, 1996). This is the opposite pattern to conventional (positive) discounting, and is referred to as negative time preference or negative discounting. Across the majority of studies the proportion of people displaying negative time preference ranges from 3 to 10%. In addition, a proportion of people do not discount hypothetical future health outcomes at all, preferring to experience better health, irrespective of delay, which is termed zero time preference (Cairns, 1992; Olsen, 1993; Redelmeier and Heller, 1993; Chapman and Elstein, 1995; Dolan and Gudex, 1995; Chapman, 1996; Chapman and Coups, 1999; Van Der Pol and Cairns, 2000; Chapman et al., 2001; Van Der Pol and Cairns, 2002, 2011).

Notably, negative and zero time preference are also observed for aversive outcomes. In choices between genuine delayed painful events, many people prefer to expedite inevitable pain (negative time preference) (Loewenstein, 1987; Berns et al., 2006; Story et al., 2013), or simply to receive less pain, irrespective of its timing (zero time preference) (Story et al., 2013). Negative time preference can be attributed to the fact that anticipating delayed pain is itself aversive, termed dread (Loewenstein, 1987), a quantity which can be minimized by choosing to “get the pain out of the way” (Loewenstein, 1987; Loewenstein and Prelec, 1991; Berns et al., 2006; Story et al., 2013). A related observation is that monetary losses tend to be positively discounted at a lower rate than monetary gains, referred to as the sign effect (e.g., Thaler, 1981; Benzion et al., 1989), which could be explained by a degree of dread for monetary losses (Loewenstein, 1987) (although could also result from differences in the shape of the instantaneous utility function for losses and gains; Loewenstein and Prelec, 1991). There is also evidence for a sign effect in the aversive domain, whereby describing delayed painful outcomes in terms of relief from a more severe pain reduced the overall degree of negative time preference (Story et al., 2013). Chapman (1996) found a sign-effect for health outcomes, whereby illness was discounted less than health improvement, to the extent that discount rates for illness were uncorrelated with discount rates for health improvement. MacKeigan et al. (1993) report a similar result for preferences over hypothetical improvements or decrements in health framed as a scenario of arthritis, finding positive discounting for health improvement and for long periods of illness, but negative discounting for fleeting illnesses.

Thus discounting behavior for hypothetical illness and health improvement closely reflects that for pain and relief. Indeed illness and health improvement are intuitively more analogous to pain and relief than to appetitive rewards and losses. Furthermore, consistent with respondents exhibiting dread for delayed illness, in an illness discounting task, individuals who perceived the illness as more severe were more likely to have negative discount rates (Van Der Pol and Cairns, 2000). Zero time preference for health and pain-related outcomes may result from the experiential nature of these outcomes; unlike money, health is an experiential commodity which cannot be saved or invested (Chapman and Elstein, 1995).

Commensurate with the prevalence of negative and zero time preference for health, discount rates for health and money are poorly correlated across individuals (Cairns, 1992; Chapman and Elstein, 1995; Chapman, 1996, 2002; Lazaro et al., 2001; Petry, 2003), and remain so even when health and monetary outcomes are utility matched (Chapman, 1996). For example, Chapman and Elstein (1995), who also included a third domain of vacations of varying duration, report an overall mean Spearman coefficient of 0.25 for the correlation between discount rates of different domains. Chapman (1996) further demonstrated that low correlations between health and monetary domains persist even when the outcomes are matched for sign as well as utility. Correlations between the two domains overall showed a trend toward being higher when matched for sign, although all correlations remained weak (Spearman *r* 0.06–0.28). In other words discount rates for illness showed marginally stronger correlations with monetary losses than with monetary gains, and discount rates for health improvement showed marginally stronger correlations with monetary gains than with monetary losses, though all such correlations are weak and most do not reach conventional levels of significance.

The low correlations between health and monetary discounting as well as the prevalence of negative and zero time preference for health and aversive outcomes demonstrate that discounting cannot be considered a universal mechanism by which all delayed events are evaluated, and appear to question any mechanistic hypothesis whereby discounting of delayed health causes unhealthy choice. It remains possible however that people represent health outcomes differently when making real health choices as opposed to responding in hypothetical health discounting tasks. In addition, discounting for either health or money may nevertheless be capable of predicting real-world health behavior, through a correlation with processes underlying health choice.

### Systematic review methods

To address the question of whether measured discount rates are capable of empirically predicting health behavior we performed systematic literature review. In order to identify studies comparing discount rates with health behavior or health status, we searched the PubMed database for full-text articles published up to January 2014, using keywords relevant to discounting together with the words “Health,” “Illness,” “BMI,” “Obesity,” “Alcohol,” “Drinking,” “Smoking,” “Drug” or “Behavior.” This search strategy yielded 104 studies. The abstracts (and where needed full text) of these were then reviewed for suitability: studies were included if they compared the results of any delay discounting paradigm to an observed health-related measure or behavior. In total 34 suitable full text articles were identified from this search. The references and citation lists of these studies were also reviewed for inclusion, yielding a further 78 suitable studies, yielding a total of 112 suitable studies (summarized in Supplementary Tables 1–5). Several of these studies have been reviewed elsewhere, chiefly in the context of addiction (Reynolds, 2006b; MacKillop et al., 2011; Bickel et al., 2012, 2014; Koffarnus et al., 2013). Here a broader range of health outcomes are reviewed. The studies are discussed below, organized by first by the modality of discounting (hypothetical health versus money or other appetitive rewards) and subsequently by the nature of the health outcomes (tobacco smoking, alcohol use, illicit substance misuse, obesity and eating behavior, preventive health behavior, risky sexual behavior and drug-taking practices or multiple health behaviors).

### Can discount rates for hypothetical health outcomes predict health behavior?

The finding that a significant proportion of individuals exhibit negative and zero discount rates for health casts doubt on the suggestion that *health* discount rates can predict unhealthy behavior. Indeed the few studies to have compared health discount rates with observed health behavior find weak or absent correlations. Four studies in our search sample examined relationships between health discounting and unhealthy behavior. Two of these tested relationships between health discounting and cigarette smoking status. Baker et al. (2003) compared discount rates for health in current and never-before cigarette smokers, finding that current smokers discounted both hypothetical improvements and decrements in health at a marginally higher rate than the never-before smokers, but this difference did not reach statistical significance. For monetary gains both hypothetical and real, current smokers delay discounted at a significantly higher rate than the never before smokers. Khwaja et al. (2007) found no significant differences between smokers and non-smokers in health discounting for health outcomes (however neither did this study find a relationship between smoking status and monetary discounting). A single study (Petry, 2003) examined discount rates for health outcomes in substance misusers, finding significantly higher discount rates for hypothetical health, money and freedom (from time spent in jail) in a group of current or previous heroin and/or cocaine users compared with a group of controls with no history of substance misuse in their lifetime. Finally, Chapman and Coups (1999) asked whether discount rates for monetary losses, described in terms of a parking fine, and for a flu-like illness, could explain uptake of free-of-charge influenza vaccinations. The respondents were future oriented, with 85% not significantly discounting the flu-like illness (i.e., showing zero time preference) and 83% not significantly discounting parking fines. Monetary time preferences were related to vaccine uptake: 45% of those with no discounting accepted the vaccine, compared with 29% of those who discounted money in the conventional manner. However, health discount rates were unrelated to vaccine acceptance. These findings suggest that hypothetical *health* discounting measures are an unreliable predictor of health behavior. The observed higher health discount rates in substance misusers compared with controls (Petry, 2003) and the trend toward higher health discounting in current cigarette smokers (Baker et al., 2003), taken together with the larger between-group differences in monetary discounting, suggest that health discounting does exhibit a weak relationship with health behavior, but is a less sensitive predictor than monetary discounting.

### Can discount rates for monetary or appetitive outcomes predict health behavior?

Discounting for hypothetical health appears to be an unreliable predictor of observed health behavior. Nevertheless many studies in our search sample demonstrate that discount rates for money or other appetitive outcomes such as food or drug rewards, correlate with health behavior or health status. The key findings are discussed below, grouped by health outcome.

#### Discount rates and self-reported health

Self-reported health is perhaps the most general health outcome measure and correlates with life-expectancy in the developed world (Idler and Benyamini, 1997). An early study (Fuchs, 1982), surveying approximately 500 adults in the US, and a more recent household survey in the Netherlands (*N* = 2300) (Van Der Pol, 2011) both found weak negative correlations between monetary discount rates and self-reported health status. A further study in a South African population (Chao, 2009) found evidence for a U-shaped relationship between subjective health and monetary discount rates, whereby those who reported “average” health had lower discount rates than those who are either very healthy or very sick. The authors suggested that this might be due to the fact that those with very poor health were in more urgent need of money to fund medical care, whereas those in excellent health were able to enjoy the benefits of immediate economic consumption, highlighting the considerable difficulties in establishing a casual pathway between discounting and health.

#### Discount rates and cigarette smoking

Monetary discount rates have consistently been shown to be higher in people who currently smoke tobacco than in non-smokers (Bickel et al., 1999; Mitchell, 1999; Odum et al., 2002; Reynolds et al., 2003, 2004, 2007a,b, 2009; Reynolds, 2006a; Fields et al., 2009; Rezvanfard et al., 2010; Stillwell and Tunney, 2012; Wing et al., 2012). A recent meta-analysis of studies comparing discount rates with addictive behaviors, (MacKillop et al., 2011) estimated a moderate and highly significant effect (Cohen's *d* = 0.57, *p* < 0.0001) across all studies comparing discount rates in smokers versus non-smokers. Monetary discount rates also correlate with smoking frequency (Epstein et al., 2003; Ohmura et al., 2005; Kang and Ikeda, 2013). In keeping with this infrequent smokers exhibit monetary discount rates intermediate between heavy smokers and non-smokers (Heyman and Gibb, 2006; Reynolds and Fields, 2012; Stillwell and Tunney, 2012 *N* = 9454; however see also Reynolds et al., 2003 and Johnson et al., 2007b for negative findings) and both smoking frequency and monetary discount rates were found to be higher in a group of young-adult smokers than in a group of adolescent smokers (Reynolds, 2004). The relationship between smoking frequency and discounting does not appear to be mediated by the acute effects of nicotine, since acute nicotine administration to non-smokers has recently been shown to have no effect on intertemporal choice behavior (Kobiella et al., 2013). However the relationship may be related to the level of nicotine *dependence* (Sweitzer et al., 2008), consistent with discounting being a state-based marker of addiction severity.

Interestingly those who have previously smoked and those who have never smoked do not significantly differ in their discounting behavior for money (Bickel et al., 1999). Furthermore in a prospective study of smoking cessation, participants were separated into a group who received an intervention program directed at reducing smoking and a control group who continued to smoke as usual. The two groups did not differ in their discounting behavior at baseline. Whilst the control group showed no changes in discounting over time, the intervention group (who overall reduced their smoking frequency) showed a significant decrease in discounting for both money and cigarettes after only 5 days into the program (Yi et al., 2008). These results strongly suggest that the state of nicotine addiction acts reversibly to increase discount rates. However there is also evidence to support the idea that discounting operates as an antecedent vulnerability marker, since monetary discount rates in smokers predict rates of smoking adoption (Audrain-McGovern et al., 2009). Monetary discount rates in smokers also predict rates of relapse within smoking cessation programs (Krishnan-Sarin et al., 2007; Yoon et al., 2007; MacKillop and Kahler, 2009; Sheffer et al., 2012; Brown and Adams, 2013), and the ability to abstain from smoking under laboratory conditions (Dallery and Raiff, 2007; Mueller et al., 2009). It seems reasonable to conclude therefore that relationships between discounting and smoking behavior are subject to both state- and trait-based influences (Odum, 2011).

Finally short-term abstinence from cigarettes increases discount rates in addicted smokers (Mitchell, 2004; Field et al., 2006; Yi and Landes, 2012). For example, Field et al. (2006) measured discount rates for hypothetical gains of money or cigarettes in a group of 30 smokers: one randomized group performed the procedures following their usual cigarette consumption, the other following a minimum of 13 h of abstinence from cigarettes. Implied discount rates for both money and cigarettes were significantly higher in the abstinence group.

In summary, monetary discount rates predict many features of smoking behavior: monetary discount rates are higher in current smokers, correlate with smoking frequency and prospectively predict the adoption of smoking and abstinence from smoking. The upward effect of nicotine cravings on discount rates and the decrease in discounting concomitant with reductions in smoking indicate that discounting is influenced by state-based environmental and motivational factors. Taken together these results indicate that relationships between discounting and smoking have both state- and trait-based components; a consideration which most likely also applies to relationships between discounting and other addictive behaviors (de Wit, 2008; Odum, 2011).

#### Discount rates and alcohol use

Monetary discount rates exhibit consistent relationships with alcohol use. A recent meta-analysis of studies comparing discount rates in persons meeting clinical criteria of an alcohol dependence syndrome with controls (MacKillop et al., 2011) demonstrated a moderate, highly significant effect (Cohen's *d* = 0.50, *p* < 0.0001). Monetary discount rates are higher in currently abstinent alcohol dependent individuals compared with non-dependent controls (Bjork et al., 2004; Mitchell et al., 2005; Boettiger et al., 2007), in early-onset as opposed to late-onset alcohol dependence (Dom et al., 2006), and correlate with the severity of alcohol dependence (Mitchell et al., 2005), as well as symptoms of an alcohol abuse disorder (MacKillop et al., 2010). Monetary discount rates have also been shown to be higher in a group with a previous lifetime diagnosis of an alcohol abuse disorder as compared with those without a lifetime history of alcohol abuse (Bobova et al., 2009).

Several studies have further linked higher discounting with relatively moderate levels of alcohol consumption. Vuchinich and Simpson (1998) demonstrated higher monetary discount rates in “problem drinkers” and also heavy social drinkers, compared with light social drinkers, suggesting a relationship between alcohol intake and discount rates even amongst those designated as social drinkers. Similarly, Field et al. (2007) found that delay discounting for alcohol positively correlated with weekly alcohol consumption (Pearson *r* = 0.31) amongst adolescents, where those in highest tertile of alcohol use had a mean weekly consumption of 23 units, while those in the lowest tertile had a mean of 3 units. Yankelevitz et al. (2012) found that implied discounting for both money and alcohol was moderately correlated with levels of alcohol use in female students (Pearson *r* = 0.43 for money and 0.41 for alcohol discounting), though no correlation was found in male students.

Amongst students, monetary discount rates appear related to adverse consequences of alcohol use (Kollins, 2003; Rossow, 2008; Dennhardt and Murphy, 2011). Kollins (2003) observed that monetary discount rates were negatively correlated with age at first using alcohol and showed a strong positive correlation with the number of times that students had “passed out” as a result of alcohol use (Pearson *r* = 0.73, *P* < 0.01) and Rossow (2008), studying a sample of 17,413 adolescents in Norway, demonstrated that high monetary discounters became intoxicated more frequently and were more likely to vomit or “pass out” as a result of drinking. Paralleling findings in previous smokers of cigarettes, previously addicted users of alcohol who have achieved long-term abstinence have discount rates intermediate between current users and controls (Petry, 2001). Finally, in an elegant field study, the discount rates of male social-drinkers on their entry to a bar prospectively predicted increases in blood alcohol level on their exit (Moore and Cusens, 2010), such that those that had steeper discounting on entry showed greater increases in alcohol level. Discount rates were not confounded by baseline intoxication, since blood alcohol level at entry to the bar did not significantly predict the baseline discount rates.

In summary, despite a minority of studies reporting negative findings (Kirby and Petry, 2004; MacKillop et al., 2007; Fernie et al., 2010), monetary discount rates (and in some studies discount rates for alcohol) show robust relationships with alcohol intake over a wide range of usage, being higher in currently dependent individuals, where they correlate with the degree of dependence, and predicting use in non-dependent individuals.

#### Discounting and illicit substance misuse

Amongst health behaviors, illicit substance misuse exhibits the most consistent relationships with discount rates. In an early study, heroin dependent individuals exhibited monetary discount rates twice those of non-drug-using controls (Kirby et al., 1999). Several other studies have also demonstrated significantly higher monetary discount rates in opioid-dependent individuals compared with controls (Madden et al., 1997; Kirby and Petry, 2004). Monetary discount rates are also higher in users of stimulant drugs such as cocaine and methamphetamine than in non-drug-using controls (Moeller et al., 2002; Coffey et al., 2003; Kirby and Petry, 2004; Heil et al., 2006; Hoffman et al., 2006; Monterosso et al., 2007; Johnson, 2012), with one study finding significantly higher monetary discount rates among individuals primarily using crack cocaine than among those primarily using heroin (Bornovalova et al., 2005). Indeed, MacKillop et al. (2011) estimated a large and highly significant aggregate effect (Cohen's *d* = 0.87, *p* < 0.0001) across studies comparing discount rates in dependent users of stimulant drugs versus controls and a moderate highly significant effect across studies comparing discount rates in opiate dependent individuals versus controls (Cohen's *d* = 0.76, *p* < 0.0001).

Consistent with nicotine abstinence studies, mild opioid deprivation in opioid dependent individuals increases discounting for money as well as heroin (Giordano et al., 2002), whereas those who have achieved longer term abstinence from heroin have lower discount rates than those currently addicted (Bretteville-Jensen, 1999; Kirby and Petry, 2004). By contrast abstinent formerly dependent cocaine users do not differ in discounting behavior from current users (Kirby and Petry, 2004; Heil et al., 2006) while Hoffman et al. (2006) found no relationship between length of abstinence and monetary discount rates in amphetamine dependent individuals. These findings suggest that either discount rates do not predict abstinence from stimulants, or that addiction to stimulants can have an irreversible effect to increase discount rates. Evidence against the former suggestion is that baseline discounting has been shown to predict the duration of abstinence from cocaine under a contingency management intervention (with low-incentives but not with high-incentives) (Washio et al., 2011).

Monetary discounting has not been consistently associated with concurrent cannabis use. Johnson et al. (2010) found that discount rates for hypothetical money in a group of marijuana dependent individuals did not differ from non-drug using controls, despite their study being adequately powered to detect any such difference (also see Stea et al., 2011). Similarly Heinz et al. (2013) found that monetary discounting did not correlate significantly with frequency of cannabis use over a 90 day period, although higher discounting was associated with younger age at first cannabis use. A recent study has shown that discount rates for hypothetical large monetary amounts ($1000) prospectively predicted abstinence outcomes amongst adolescents undergoing treatment for marijuana dependence (Stanger et al., 2012), more recent studies have (Heinz et al., 2013; Peters et al., 2013) found that discount rates did not predict response to a similar intervention in adults.

Finally studies have demonstrated an additive effect of smoking and alcohol use on discounting (Moallem and Ray, 2012; see also Andrade et al., 2013) but not of smoking and other forms of substance misuse (Businelle et al., 2010), and the combination of gambling problems and substance misuse appears highly predictive of impulsive choice (Petry and Casarella, 1999; Petry, 2001; however see Ledgerwood et al., 2009). In summary, with the exception of cannabis use, monetary discount rates consistently show strong correlations with the use of illicit substances.

#### Discounting, obesity, and eating behavior

Researchers have examined relationships between obesity and discounting for both food and money outcomes, citing similarities between eating behavior and addiction. Obese children have been shown to choose immediate over delayed edible rewards more often than normal weight children, though the effects were small and not found for non-food rewards (Johnson et al., 1978; Bonato and Boland, 1983). Notably the ability to delay gratification for food rewards at aged four predicts the likelihood of being overweight at aged 11 (Seeyave et al., 2009). In addition, cross-sectional studies have examined links between measures of monetary discount and Body Mass Index (BMI) in adults, with mixed findings (Epstein et al., 2003; Borghans and Golsteyn, 2006; Nederkoorn et al., 2006; Reimers et al., 2009; Ikeda et al., 2010). In a large sample from the Netherlands financial proxies for the discount rate, for example reported under-saving or excessive expenditure, were significantly correlated with BMI, however measured discount rates themselves were not (Borghans and Golsteyn, 2006). Similarly, Ikeda et al. (2010) found that BMI was positively correlated with a survey measure of procrastination, but showed no correlation with monetary discount rates in a sample of 2987 Japanese adults. In group comparisons, obese women have been shown to exhibit significantly higher discount rates than healthy weight women (Weller et al., 2008), and people who smoke cigarettes who are also obese to exhibit higher rates than non-obese smokers (Fields et al., 2011). Davis et al. (2010) found that obese women with a binge-eating disorder, but not obese women without binge-eating disorder, had significantly higher monetary discount rates than normal weight women. It has been suggested that sensitivity to food rewards interacts with delay discounting, in support of which high discount rates predict palatable food intake amongst normal weight women who find palatable foods highly rewarding (Rollins et al., 2010), an effect which has been replicated in obese and overweight women (Appelhans et al., 2011). More recently Kulendran and colleagues found significantly higher monetary discount rates in obese adolescents compared with normal-weight adolescents (Kulendran et al., 2013a), and demonstrated that monetary discount rates in obese adolescents decreased over the course of a residential obesity intervention (Kulendran et al., 2013b). Taken together these studies suggest an emerging relationship between discounting and weight status, although further work is clearly required to establish whether particular aspects of eating behavior, such as caloric intake, or eating frequency show relationships with discounting.

#### Discount rates and preventive health behavior

While some studies find relationships between discounting and preventive health behaviors, the findings are less consistent than for addictive behaviors. As described above, Chapman and Coups (1999) asked whether discount rates for monetary losses, as well as for a flu-like illness, could explain uptake of influenza vaccinations, with the finding that time preferences for money, but not illness, were related to vaccine uptake. In a later study (Chapman et al., 2001) monetary discounting showed an absent or very weak correlation with compliance with anti-hypertensive or cholesterol lowering medication. Similarly, a meta-analysis (Chapman, 2005) of 16 existing studies, including those described above, found no significant correlation between discounting and preventive health behavior (Mean Pearson *r* = 0.04, 95% *CI* = −0.01, 0.09). These studies suggest that in the population as whole preventive health behaviors show little or no relationship with discounting. However, two subsequent studies suggest that a subset of the highest discounters diverge from the rest of the population in their patterns of preventive health behavior. Firstly, Axon et al. (2009), studying 422 hypertensive adults, found that those in the highest quintile of monetary discount rates reported that they would be less likely to alter their diet and exercise plans to improve their future health. The highest discounters were not however significantly less likely to check their blood pressure or to follow their doctors' plans, as assessed by self-report. Secondly, Bradford (2010), analyzing discounting in 978 adults, found that for high discounting women the implied probability of attending mammography was reduced by 15.30% over the preceding 2 years and high discounting men had significantly lower rates of prostate examination (probability reduction 8.31%). The influence of discounting on attendance for cervical cancer screening was marginally significant. Across gender, high discounters were significantly less likely to have attended the dentist (probability reduction of 24.8%) or to have had any cholesterol testing (probability reduction 12.38%) or any influenza vaccination (probability reduction 11.05%) over the preceding 2 years. Additionally, high discounters were significantly less likely to be non-smokers or to have undertaken weekly vigorous activity. These studies suggest that monetary discount rates might be a useful tool for identifying groups at risk of failing to engage in preventive health practices.

#### Discount rates and risky sexual behavior or drug-taking practices

Convergent evidence associates high monetary discount rates with behaviors that increase the risk of contracting sexually-transmitted or blood-borne viral infections. Individuals infected with hepatitis C exhibit higher rates of discounting than controls (Huckans et al., 2010), although the direction of causality cannot be established from this study. Higher discount rates are associated with needle-sharing amongst heroin users (Odum et al., 2000). Dierst-Daviese t al. (2011) found that a sample of homeless, men who abused substances and had sex with men, had higher discount rates than a control sample of men, deemed to be at lower risk of HIV, who had sex with men however had stable housing and did not abuse substances. Finally, Chesson et al. (2006) found relationships between monetary discounting and a spectrum of sexual behaviors and outcomes in a combined sample of university students and adolescents attending clinics (*N* = 1042). For example, adolescents with higher discount rates were more likely to have had sexual intercourse before age 16 years, to have contracted gonorrhea or chlamydia, or to have become pregnant.

#### Discount rates and multiple health behaviors

Two studies in our search sample compared discount rates with a broad range of health behavior. Firstly Daugherty and Brase (2010), collected data from 467 undergraduates on an inventory of health behaviors, namely tobacco, alcohol and drug use, number of visits to a doctor or dentist in the past year, exercise frequency, eating breakfast, seat-belt use when in a vehicle, motorbike or bicycle helmet use, and the use of sunscreen. They found that, in a two-step hierarchical regression analysis, a combination of delay discounting for hypothetical money and survey measures of time perspective explained a significant proportion of the overall variance in health behavior over and above the combination of the respondents' gender and their personality type (Costa and McCrae, 1990). At the level of predicting individual behaviors, the improvement in model fit achieved by adding the time preference measures at the second step was small (the largest improvement in R^2^ was 0.05) but significant for all the behaviors above except helmet-wearing. Secondly Melanko and Larkin (2013) analyzed data from 72 young adults who performed both a discounting task with real monetary rewards and a hypothetical monetary discounting task as well as completing a Healthy Lifestyle Questionnaire (Corbin et al., 2006), assessing a variety of health behaviors, including smoking, alcohol use, substance misuse, physical activity, nutrition, avoiding destructive habits or practicing safe sex. In a hierarchical multiple regression, implied monetary discount rates for real rewards emerged as a significant predictor of the overall variance in health behavior. With regard to specific health behaviors, discounting for real monetary rewards emerged as a significant predictor of only smoking and nutrition scores. Notably however neither Daugherty and Brase (2010) nor Melanko and Larkin (2013) separated individuals by their level cigarette, alcohol or drug use. As a result the observed relationships between discounting and other behaviors may have been confounded by the effects of these addictive behaviors to increase impulsivity in other domains.

### Conclusions: monetary discounting predicts unhealthy behavior

Taken as a whole, the studies reviewed here support the hypothesis that high discount rates for money, and in specific instances food or drug rewards, are associated with many unhealthy behaviors. Furthermore the effect sizes reported compare favorably to existing social cognitive models of health behavior (Conner and Norman, 2005), establishing high discounting as a reliable correlate of unhealthy choice.

The majority of studies reviewed above are cross-sectional and are therefore indeterminate as to whether high discounting antecedes unhealthy behavior, or vice versa. Two longitudinal studies reviewed here demonstrate that monetary discounting can prospectively predict onset of unhealthy behavior or relapse after health behavior change (Yoon et al., 2007; Audrain-McGovern et al., 2009). However, there is also considerable evidence that discounting is influenced by state-based factors (Koffarnus et al., 2013). In addition, for addictive behaviors, the severity of addiction is positively correlated with discounting (for example Mitchell et al., 2005; Sweitzer et al., 2008; MacKillop et al., 2010), and discount rates have been shown to decrease following behavior change (Landes et al., 2012). These observations combine to suggest that discounting can be viewed as a concurrent marker of the extent of unhealthy behavior, rather than exclusively as an anteceding risk factor. Consistent with either interpretation, a growing number of studies have shown that monetary discounting predicts response to behavior-change interventions (Dallery and Raiff, 2007; Krishnan-Sarin et al., 2007; MacKillop and Kahler, 2009; Mueller et al., 2009; Washio et al., 2011; Sheffer et al., 2012; Stanger et al., 2012; Brown and Adams, 2013). Thus discounting has clear predictive utility and may allow health-behavior change interventions to be tailored to benefit at-risk groups. In addition, interventions may be targeted at modifying the cognitive mechanisms associated with discounting, which are assumed to contribute to unhealthy behavior. For example working memory training has been shown to both reduce discount rates and modify addictive behavior (Bickel et al., 2011).

However, in order for discounting to be upheld as a *mechanism* for unhealthy choice, the features of unhealthy behavior must also be consistent with the predictions of a particular model of discounting. We test this by examining the proposal that hyperbolic discounting can explain goal-incongruent unhealthy action. We conclude that hyperbolic discounting on its own has explanatory shortcomings, and might be usefully supplemented by a broader conceptual framework.

## Does hyperbolic discounting explain goal-incongruent unhealthy actions?

**Figure 1:**
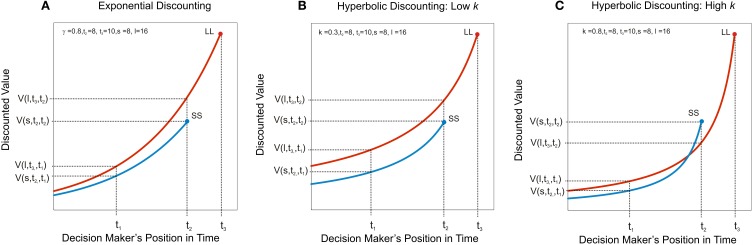
**Hyperbolic discounting predicts myopic preference reversal**. Discounted value, *V*(*A, t*, τ) under three discount functions is plotted of as a function of the decision maker's position in time, τ, where *A* is the magnitude of the outcome (its instantaneous utility) and *t* the time at which it is due to be delivered. A larger-later reward, LL, of magnitude, l, is due to be received at *t*_3_ and a smaller-sooner reward, SS, of magnitude, s, is due to be received at *t*_2_. **(A)** Exponential discounting. The decision-maker has consistent preferences, such that the ratio of the value of two rewards is constant irrespective of how far away the options are in time; in this case the decision-maker always prefers the larger later reward (i.e., *V*(*l*, *t*_3_, τ) > *V* (*s, t*_2_, τ) for all τ < *t*_3_). **(B)** Hyperbolic discounting with a low discount rate. The ratio of the value of two rewards is no longer constant as a function of τ. The hyperbolic discount rate, *k*, governs the steepness of the curvature. Here, where *k* is low (*k* = 0.3) the larger later reward is still preferred, even when the smaller sooner reward is immediately available. **(C)** Hyperbolic discounting with a high discount rate. At *t*_1_, when both rewards are distant, the larger later reward is preferred, i.e., *V*(*l, t*_3_, *t*_1_) > *V* (*s, t*_2_, *t*_1_), however the smaller sooner reward becomes increasingly desirable as it approaches in time, such that at *t*_2_, the immediately available smaller reward is preferred, i.e., *V*(*l, t*_3_, *t*_2_) < *V*(*s, t*_2_, *t*_2_). This prediction of hyperbolic discounting has been proposed to underlie the observation that individuals make far-sighted plans for the distant future, but often renege on those plans in favor of short-term gratification when the future arrives.

The observation that hyperbolic functions consistently provide excellent fits to choices between delayed rewards has led to the suggestion that hyperbolic discounting may be a guiding computational principle of intertemporal choice (Ainslie, 1975, 2001). In particular, since the curvature of the hyperbolic function predicts myopic preference reversals (Figure 1), hyperbolic discounting has been widely proposed as a an explanation for impulsive reward-seeking at the expense of long-term plans (for example Laibson, 1997; Ainslie, 2001; Angeletos et al., 2001; Bickel et al., 2007, 2012). Taken together with the observation that hyperbolic discount rates correlate with many forms of unhealthy behavior (Supplementary Tables 1–5 indicate which of the above studies measured hyperbolic rates), hyperbolic discounting proffers to explain goal-incongruent unhealthy action. In this section we examine this hypothesis in more detail.

### Evidence for and against hyperbolic discounting

A key prediction of hyperbolic discounting is that, where a smaller-sooner reward is preferred over a larger-later reward, adding sufficient delay to both sooner and later options ought to shift preference toward the larger-later reward (see Figure 1). Several studies have demonstrated evidence for this in conventional monetary discounting tasks (for example Green et al., 1994; Kirby and Herrnstein, 1995). Such preference reversals have also been demonstrated in choices with health-relevant outcomes. For example, Read and Van Leeuwen (1998) asked people whether they would prefer to receive in 1 week's time either a healthy snack (such as a piece of fruit) or an unhealthy snack (such as a chocolate bar). The same individuals were followed up and 1 week later they were offered an immediate choice between a healthy and an unhealthy snack. Respondents chose healthy snacks more frequently when the choice was made in advance compared to when the snacks were immediately available. If healthy behavior is considered to carry larger-later health rewards, this result is consistent with the hyperbolic discounting of delayed health.

Despite the above findings, subsequent studies have challenged the hyperbolic model of preference reversal (Read, 2001; Read and Roelofsma, 2003; Sayman and Öncüler, 2009; Kable and Glimcher, 2010; Read et al., 2012). For example Kable and Glimcher (2010) found that discount functions based on a choice set in which all options were delayed by a fixed amount had the same hyperbolic curvature as those based on the same choice set in which the sooner option always occurred immediately: in contrast to conventional hyperbolic discounting, in which all outcomes are evaluated relative to the present time, this “as-soon-as-possible” function does not predict impulsive preference reversals. Additionally, several longitudinal studies have tested the predictions of hyperbolic discounting in real-time using monetary outcomes, with mixed findings (Ainslie and Haendel, 1983; Sayman and Öncüler, 2009; Read et al., 2012). The earliest of these studies found support for the preference reversals predicted by hyperbolic discounting. In this study Ainslie and Haendel (1983) asked participants on a Monday to choose between smaller amount of (hypothetical) money on to be received on Friday and larger amount to be received the following Monday. Participants were offered the choice again on the Friday, this time between receiving the smaller amount (for real) immediately or the delayed amount on the coming Monday. Consistent with hyperbolic discounting, the most common pattern was a preference for the larger-later amount when choices were made in advance, but for the smaller sooner amount when this was immediate. However subsequent studies have not replicated this finding. (Sayman and Öncüler, 2009) found the opposite result using a design similar to that of (Ainslie and Haendel, 1983). Furthermore a recent study performed over several weeks using real monetary rewards showed that preference reversals toward choosing smaller-sooner amounts (that is, in the direction predicted by hyperbolic discounting) were not significantly more common than those in the opposite direction (Read et al., 2012). Importantly this was the case despite the participants displaying hyperbolic discounting in conventional “cross-sectional” choices.

In summary, the preference reversals of the form predicted by conventional hyperbolic discounting have hitherto not been consistently demonstrated with monetary outcomes. This suggests that the preference reversals underlying health-related choices (such as those in Read and Van Leeuwen, 1998) may result from peoples' inability to predict in advance the impact of motivational and environmental states on their future decision-making.

### Goal-incongruent actions often result from state changes

The suggestion that intention-action discrepancies in health choice result exclusively from hyperbolic discounting (Ainslie, 2001) can also be questioned. Everyday experience suggests that people often abandon long-term plans in favor of immediate reward in response to environmental cues or changes in internal motivational state; for example, one might plan to abstain from eating dessert as part of a diet plan, but find it harder to resist when presented with a piece of cake (see for example Allan et al., 2010). Loewenstein (1996) has proposed that motivational drives and cues which elicit them, rather than hyperbolic discounting, are responsible for impulsive preference reversal. This idea is supported by evidence; for example, relapses in drug-taking behavior following abstinence commonly occur after exposure to a previous drug-taking environment (O'Brien et al., 1998). Hyperbolic discounting, to the extent that it applies as a model for preference reversal, may in some instances be sufficient to explain these cue-triggered behaviors, since cues provide information about the *timing* of outcomes, thereby signaling that reward is at hand. However, hyperbolic discounting does not appear necessary to explain these state-dependent influences. For example, in a study of analgesic preferences for childbirth (Christensen-Szalanski, 1984), women asked roughly 1 month in advance of labor preferred to avoid invasive spinal anesthesia in favor of less invasive but less effective pain relief methods, however during active labor women frequently reversed preference and opted for anesthesia. These findings are easily explained by an increase in the marginal utility for anesthesia during the painful state, which was not accurately predicted in advance, without reference to hyperbolic discounting.

In summary therefore not all forms of health-related preference reversal are consistent with hyperbolic discounting, and many preference reversals occur in response to changes in motivation or environmental states. Taken together with the lack of clear longitudinal evidence for the myopic preference reversals predicted by hyperbolic discounting, this suggests that any generative model of intertemporal health choice ought to be expanded beyond hyperbolic discounting alone to account for the effects of environmental cues. At best hyperbolic discounting alone provides no explicit framework either for incorporating the motivational information provided by environmental cues, or for how this information becomes associated with cues through learning. Existing models for these influences have not aimed to provide a mechanistic level of interpretation (Loewenstein, 1996). The following discussion advances a mechanistic framework based on the principles of reinforcement learning for understanding the effects of environmental cues on intertemporal health choice. Key to this account is the notion that cues previously associated with rewarding actions can trigger goal-incongruent habits, leading to preference reversal even in the absence of hyperbolic discounting. A full exploration of learning is beyond the scope of this review and we therefore restrict ourselves to the effects of cues after learning has taken place.

## A reinforcement learning approach to cue-triggered preference reversal

Reinforcement learning provides an approach to understanding intertemporal choice. Models of reinforcement learning posit that action control proceeds by estimating the expected value of ensuing reward over series of temporally connected future states, encapsulated in a state-action value function (Sutton and Barto, 1998); such models are therefore well placed to incorporate changes in state on choice behavior. Attempting to optimize value in changing environments can be considered a trade-off between flexibility in rapidly incorporating new information and the efficient use of past experience (Daw et al., 2005). This trade-off is embodied by two methods of learning action-value: a rather rigid, but computationally lean method, referred to as model-free, and a flexible, planning method capable of simulating future possible outcomes, often referred to as model-based (Gläscher et al., 2010; Daw et al., 2011; McDannald et al., 2011; Daw, 2012; Wunderlich et al., 2012; Dolan and Dayan, 2013). These systems reflect an established distinction in psychology between deliberative and automatic processes (Evans and Stanovich, 2013), but endow this with a normative and explicitly computational basis (Daw et al., 2005).

A model-based decision-maker is generally assumed to search through the possible future states consequent on each action. Model-based decision-making corresponds to the definition of “goal-directed” behavior in animal learning experiments as rapidly sensitive to changes in outcome value or the contingency between response and outcome (Colwill and Rescorla, 1986; Dickinson and Balleine, 1994; Balleine and Dickinson, 1998; Domjan, 2003). A model-free decision-maker, by contrast, through a gradual integration of outcome values encountered through experience, assigns a scalar estimate of long-run future value to taking an action in a particular state, without explicitly representing the corresponding future state of the world. The resulting cached action values are relatively insensitive to immediate changes in the outcomes. Instrumental behavior is initially goal-directed (model-based), but becomes increasingly model-free with learning, such that actions eventually become insensitive to changes in the value of the outcome, acquiring the characteristic of habits (Dickinson et al., 1995; Ouellette, 1998; Neal, 2006). This bears direct analogy to economic models of habit formation, which modify the instantaneous utility function to depend on past consumption (Becker and Murphy, 1988).

The differential engagement of these two systems has the potential to explain the environmental dependence of the preference reversals which underlie many forms of unhealthy behavior. While steep temporal discounting over the model-based valuation of future health would be expected to encourage the initiation of unhealthy behavior, with repetition, unhealthy behavior is likely to become increasingly model-free, or habitual. At this stage, even if the decision-maker re-evaluates their goals in favor of making healthy choices, cached action values will continue to encourage unhealthy choice in response to relevant environmental cues, leading to apparently impulsive preference reversals (intention-action discrepancies).

### Environmental cues can trigger goal-incongruent habits

**Figure 2:**
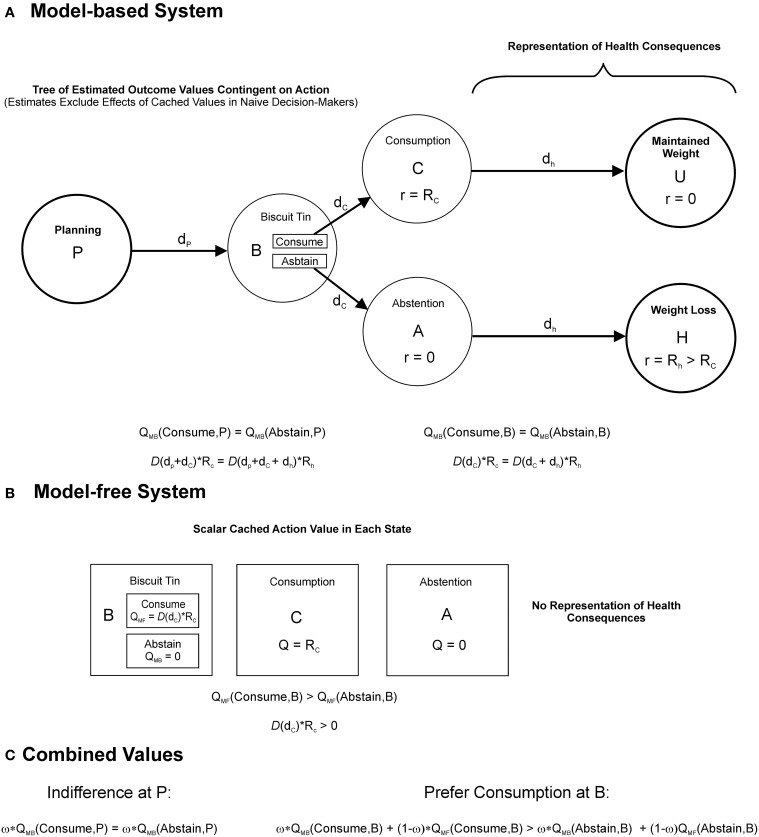
**Interactions between model-based and model-free decision-making**. Action values for a hypothetical agent, a person following a diet-plan, deciding whether or not to consume biscuits when presented with a cue, the biscuit tin. The agent's choice combines model-based and model-free value. **(A)** A decision-tree (semi-Markov state-space) represented by the model-based system when the agent considers the decision from state, *P* at a time interval, *d_p_*, in advance of encountering the biscuit tin, denoted by state B. Alternative courses of action at *B*, to *consume* or to *abstain*, are evaluated by searching through the tree of future possibilities. The choice to *consume* is followed after a short delay, *d_c_*, with a food reward, *R*_*c*_, associated with consumption, denoted by the state C, followed after a longer delay, *d*_*h*_, by the maintenance of current body weight, denoted by the unrewarded state, *U*. The choice to abstain is followed after delay, *d*_*c*_, by the unrewarded state *A*, followed after delay, *d*_*h*_, by a health benefit with reward, *R*_*h*_, in the form of weight loss. The agent is naïve to the parallel effects of model-free learning when computing these reward estimates. Model-based action values, *Q*_*MB*_, are given by the sum of future rewards following each action, discounted according to a function, *D*(*t*), assumed to be exponential and identical across both controllers. The equations below indicate that the model-based system in this instance is indifferent between consuming and abstaining at both P (left hand equation) and B (right hand equation). **(B)** Cached values stored by the model-free system, which reflect the result of prior experience with the outcomes. Neither the outcomes themselves, nor the transitions between them, are explicitly represented. Similarly, because the distant health consequences have never been experienced, they do not influence the model-free *Q*-values, *Q*_*MF*_. As a result the model-free system prefers consumption at state B. **(C)** Model-based and model-free values are assumed to combine according to a weighted average, governed by the parameter, ω. At *P*, where model-free values have no influence, the agent is indifferent between consuming and abstaining. In the presence of the biscuit tin at *B* however the additional influence of model-free (cached) values induces a preference for consumption.

Once a person has initiated an unhealthy behavior, they may later change their goals and form the intention to abstain from that behavior. For example, a person who smokes might decide to quit after being diagnosed with lung disease. However, if sufficient learning has taken place, the behavior might nevertheless be maintained as a stimulus-response habit under the dominant influence of cached action values. Thus, the smoker might find it particularly hard to resist when he or she spies the cigarette packet. As we outline below, the goal-incongruent influence of cached (habitual) action values can produce preference reversal, without invoking hyperbolic discounting. Furthermore, preference reversal can result even if each system in isolation exhibits exponential discounting and discounts the future at the same rate, a crucial distinction from dual-process models of quasi-hyperbolic discounting (Laibson, 1997; Angeletos et al., 2001; McClure et al., 2004; Bickel et al., 2012; Koffarnus et al., 2013). To demonstrate this formally, consider a decision-making agent for whom overall action value is a weighted average of the value from each controller, where both systems discount the future exponentially with a per period rate, γ (γ is the conventional symbol for the discount rate in reinforcement learning approaches; its meaning is equivalent to that of δ in Equation 1). Say, for example, the agent is a person following a diet plan who is choosing whether or not to consume a calorific biscuit when faced with a cue, the biscuit tin. A simplified (semi-Markov) state space for this decision is depicted in Figure 2A. State B represents the presence of the biscuit tin. Consuming biscuits leads after a short delay, *d_c_*, to state C, which carries reward, *R_c_*, and after a longer delay, *d_h_*, to maintaining current weight, for simplicity here assigned a reward value of zero. Abstaining from biscuits leads, via the unrewarded state, *A*, to a health benefit in the form of weight loss, *R_h_*, after delay *d_h_*. Notably this is a radical simplification of reality. It is assumed that, while the model-based system is capable of making such simplifications based on declarative knowledge, the model-free system cannot, and has never experienced the health consequences. As a result, the model-free system has learned the values of each action in state B (termed “*Q*-values”) based solely on the reward previously provided from consumption (Figure 2B). It is assumed that the model-based system is initially naive to these cached values. Consider that the agent, after learning, is asked to make their decision when situated in state P, at some time delay, p in advance of state B, where cached values have no influence, and that here they are indifferent between indulging and abstaining, that is to say that the model-based value, *Q*_*MB*_, of consumption is equal to that of abstention:

(3a) $$Q_{MB}(\text{Abstain},\;P)=Q_{MB}(\text{Consume},\;P)$$

Given by:

(3b) $$R_{h}\cdot \gamma^{p+c+h}=R_{c}\cdot \gamma^{p+c}$$

Which simplifies to:

(3c) $$R_{h}\cdot \gamma^{c+h}=R_{c}\cdot \gamma^{c}$$

On reaching B, the presentation of the biscuit tin, cached action values are also “brought online,” incrementing the benefit of indulging, such that:

(4a) $$Q_{\text{COMBINED}}\left(\text{Consume},\;B\right)=R_{c}\cdot \gamma^{c}\cdot \omega +R_{c}\cdot \gamma^{c}\cdot (1-\omega )$$

Given by:

(4b) $$Q_{\text{COMBINED}}=\;Q_{MB}\cdot \;\omega +Q_{MF}\cdot \;(1-\omega )$$

(4c) $$Q_{\text{COMBINED}}\left(\text{Abstain},\;B\right)=R_{h}\cdot \gamma^{c+h}\cdot \omega$$

And therefore, by (3c):

(5) $${\text{R}}_{h}\cdot \gamma^{c+h}\cdot \omega \leq R_{c}\cdot \gamma^{c}\cdot \omega +R_{c}\cdot \gamma^{c}\cdot (1-\omega )$$

Predicting a preference for indulging for ω < 1 (Figure 2C). Therefore the presentation of the biscuit tin brings about a preference for sooner consumption. In economic terms, environmental cues such as the biscuit tin might be viewed as updating the utility of the immediately available option, by providing (previously inaccessible) information from prior experience.

The interplay between model-based and model-free systems in the account above bears some similarity to existing dual-systems models of intertemporal choice, which posit a deliberative planning system in opposition with an impulsive system. However, while the former are often mapped onto quasi-hyperbolic models of discounting (McClure et al., 2007, 2004), which combine two exponential discount functions with differing rates, here the two systems may share the same discount rate. Dynamic inconsistency can then result from the different sources of information available to either controller (also see Dayan et al., 2006). In particular, the state-dependent valuations of the cached system can explain why real-world preference reversals occur in response to learned cues and, unlike existing quasi-hyperbolic accounts, why these preference reversals become more prominent with the formation of habits. In addition, unlike existing dual-process accounts, reinforcement learning models can explicitly model the learning process generating incongruent preferences. (A direct treatment of learning is beyond the scope of this review).

Exponential discounting is used here to illustrate that hyperbolic discounting is not necessary to predict preference reversals, although clearly hyperbolic discounting is more consistent with cross-sectional intertemporal choice data than exponential discounting. The framework above could readily incorporate hyperbolic discounting, and several authors have demonstrated reinforcement learning models which produce hyperbolic discounting (Daw and Touretzky, 2000; Tsitsiklis and Van Roy, 2002; Kalenscher and Pennartz, 2008; Kurth-Nelson and Redish, 2009; Alexander and Brown, 2010).

### Model-based and model-free interactions in addiction

The relative contributions of model-based and model-free strategies might in part explain why discount rates correlate particularly strongly with addictive behaviors (Keramati et al., 2012; Lucantonio et al., 2014). Steep discount rates would putatively favor initial goal-directed drug-seeking behavior (as with other forms of unhealthy behavior). The high rewards provided by substances of abuse might then lead to rapid habitization of drug taking behavior by comparison with other repeated behaviors (Everitt and Robbins, 2005; Everitt et al., 2008; Lucantonio et al., 2014), effectively binding impulsive individuals to their initial choices. In addition, repeated choice of immediately available rewards by individuals with high discount rates would be expected to lead to these individuals acquiring habits more rapidly (by more reinforced choices). In support of this, animal studies of addiction demonstrate that rats bred to exhibit steeper delay discounting more rapidly acquire compulsive self-administration of cocaine than their low discounting counterparts (Belin et al., 2008). Finally, chronic addiction may further shift responding toward model-free control (Keramati et al., 2012), in part by damaging frontal cortical areas on which model-based valuations are thought to depend (Rogers and Robbins, 2001; Gläscher et al., 2010; Camchong et al., 2011; Smittenaar et al., 2013), further decreasing the capacity to exert model-based control over goal-incongruent habits. Although these mechanisms most likely play in a role in addiction, there is an ongoing debate as to their precise contribution, and in particular the interplay between habitual mechanisms and classical (Pavlovian) conditioning in addictive disorders (Everitt and Robbins, 2005).

## Future directions

The studies reviewed here indicate that discounting is a promising predictor of health behavior, however hyperbolic discounting is challenged as an explanation for the discrepancy between intentions and actions in health choice, and a framework based on the trade-off between model-free and model-based action control appears better placed to incorporate the influences of environment and learning. Nevertheless the study of myopic health-related decision-making remains nascent. Further work is required firmly establish discounting as a predictive tool, to extend the measurement framework and to develop novel interventions capable of reducing goal-incongruent health choice.

### Discounting as an individual difference measure

The endeavor to predict and understand health behavior through comparison with discounting measures forms part of a wider paradigm to characterize individual differences in field behavior using decision-making tasks (Montague et al., 2012). A question relevant to this endeavor is the extent to which discounting can be considered as a either a personality trait or a state variable (de Wit, 2008; Odum, 2011; Bickel et al., 2012). Personality traits are defined as stable and enduring characteristics, reflecting a general tendency to respond in a given manner under given circumstances and can be seen to represent persistent patterns of internal states (see Costa and McCrae, 1990). State variables by contrast vary over a shorter time scale, such that their rank ordering between individuals may be altered with changes in the motivational state of the respondents and/or the elicitation conditions (Kraemer et al., 1993).

Several pieces of evidence reviewed here demonstrate that discounting has a state-based component. Firstly studies comparing the discounting of hypothetical health with that of money find that discounting varies with the domain and valence of the outcome, in a manner that changes the rank ordering of discount rates between individuals. Secondly discount rates amongst substance misusers are greater in a state of drug-craving than in a drug-sated state. Thirdly, even under conditions of drug-satiety, addiction appears to be associated with a reversible increase in discounting. These findings are supported by a wealth of additional evidence showing that discounting can be manipulated through contextual framing (see Koffarnus et al., 2013 for a review). Indeed from a normative perspective it is sensible for agents to adjust their tolerance of delay to match environmental conditions; for example steep discounting is adaptive in an environment where delayed rewards are highly uncertain to be received (see Lahav et al., 2011).

However there is also evidence that discounting has attributes of trait variable. The test-retest reliability of monetary discounting is substantial at intervals of up to 1 year (Pearson *r* = 0.71; Kirby, 2009) and across different elicitation methods (Odum, 2011). Furthermore, whilst monetary discounting is poorly correlated with hypothetical health discounting across individuals monetary discount rates are strongly and significantly correlated with other forms of appetitive outcome, such as the discounting of cigarettes for cigarette smokers, the discounting of heroin for opioid-dependent outpatients and the discounting of food amongst college students (Odum, 2011; Pearson *r* = 0.93; *p* = 0.0007 for money versus the mean of all other outcomes). There is also evidence that discounting is heritable (see MacKillop, 2013 for a review). A recent longitudinal twin study estimated the heritability of delay discounting in adolescence at up to 50% (Anokhin et al., 2011), rats and mice can be bred to exhibit greater degrees of delay discounting (e.g., Anderson and Woolverton, 2005; Belin et al., 2008), and steeper discounting in humans is associated with specific polymorphisms related to dopamine signaling (Eisenberg et al., 2007). Also commensurate with discounting as an enduring trait, steeper discounting is associated with lower socio-economic status (e.g., Bradford, 2010; Anokhin et al., 2011). In summary, discounting for appetitive outcomes has features of a trait marker.

Trait-level differences in discounting can be viewed as long-term adaptations to prevailing environmental conditions, shaped either through learning or inheritance (this notion is consistent with a branch of evolutionary theory termed Life History Theory; Del Giudice et al., 2013; Del Giudice and Ellis, 2014). An important direction for future research will be to examine the relative contributions of genes and childrearing environment to discounting. The study of self-regulation in developmental psychology has adopted this approach; for example children who experience emotionally close, sensitive, and responsive caregiving have been found to exhibit higher levels of self-regulation (Belsky et al., 2007), and Berry et al. (2013) find evidence that self-regulation ability appears to be more sensitive to early childcare experiences in a group with a particular dopamine receptor polymorphism. Furthermore low childhood self-regulation has been prospectively related to poorer health outcomes later in life (for example Francis and Susman, 2009; Seeyave et al., 2009; Moffitt et al., 2011). Future research into delay discounting would benefit from a similar developmental perspective to better understand the origins of trait-level individual differences.

In conclusion discount rates are far from immutable, and are sensitive to environmental and motivational conditions. However, discounting for appetitive outcomes is stable across individuals when measured under similar conditions, is partly heritable and is associated with a range of similar constructs, and as a result has the potential to provide an endophenotype which mediates between genetic influences, more fundamental neuro-computational processes and maladaptive patterns of impulsive behavior in the real-world (MacKillop, 2013). Further work is required to more completely characterize the relationships between these levels of analysis. We have proposed that discounting is best considered within a broader framework for understanding choice between temporally extended outcomes, based on the theory of reinforcement learning. We have shown how the interaction between model-based and model-free value estimates may contribute to real-world instances of goal-incongruent unhealthy choice. However several important questions remain largely unanswered. For example, can the balance of model-based versus model-free control be measured, and can such measures be used to predict health-related behavior? Is there a trait component to this balance? What is the relationship between model-based control and measured discount rates, or related metrics of self-regulation? We briefly address these questions in turn below.

### Measuring model-based and model-free interactions

One approach to measuring the interaction between model-based and model-free decision-making is to directly observe the acquisition of habitual behavior through repeated training on a given laboratory task. Here, the rate of acquisition of habitual responding may offer a novel measure for predicting field behavior. Using this approach, outcome-insensitive habits have been demonstrated in humans (Tricomi et al., 2009), providing a behavioral counterpart to studies of habit formation which measure subjective automaticity (Lally et al., 2010). An important aim for future studies will be to examine habitual or cue-triggered preference reversals in real-time. Along these lines, subjective measures of habitual automaticity in relation to smoking behavior have been shown to predict goal-incongruent smoking-related responses (Orbell and Verplanken, 2010). However, observing habit learning directly is time-consuming. Recent human studies (Gläscher et al., 2010; Daw et al., 2011; Eppinger et al., 2013; Smittenaar et al., 2013) have used a paradigm with a probabilistic tree structure which separates model-free and model-based control, before habitization has taken place, depending on whether respondents incorporate the transition structure of the task into their learning (model-based) or learn solely based on the reinforcement obtained in each discrete state (model-free). Humans performing this task generally exhibit some combination of the two modes of control, and the relative contribution of the two strategies may provide a novel behavioral marker. Further studies are required to establish the longitudinal stability of these measures, and whether they have a trait component, as well as to examine their relationship with habitual behavior in the field.

### Relationships between model-based control, discounting and self-regulation

Responding on discounting paradigms cannot easily be considered habitual, and most likely requires model-based processes. Nevertheless, we propose that directly representing outcomes during choices on discounting paradigms, rather than relying on a low-level tradeoff between amount and delay, is likely to be associated with more future-oriented responses. In line with this suggestion, mentally simulating future outcomes decreases measured discount rates (Peters and Büchel, 2010) and lesioning neural structures on which this simulation process depends, such as the hippocampus (Hassabis et al., 2007; Johnson et al., 2007a; Schacter et al., 2008) increases discounting (Mariano et al., 2009). Furthermore, existing studies suggest that the choice of delayed rewards, model-based control and working memory engage overlapping neural substrates: neuroimaging studies have found that the dorsolateral prefrontal cortex (dlPFC) is activated in both model-based learning (Gläscher et al., 2010), and in choosing delayed rewards on intertemporal choice paradigms (McClure et al., 2004, 2007), while disrupting this area (using either transcranial magnetic stimulation or transcranial direct current stimulation) both decreases model-based behavior (Smittenaar et al., 2013) and increases temporal discounting (Hecht et al., 2013). A recent study has also demonstrated that in younger adults, but not in older adults, a greater degree of model-based behavior is associated with higher working memory capacity (Eppinger et al., 2013). The finding that working memory training decreases discounting among substance misusers (Bickel et al., 2011) is especially interesting in this regard. A possible unifying interpretation is that explicitly representing the future consequences of action, a process associated with model-based decision-making, produces more future-oriented choice and hence lowers discount rates (see also Peters and Büchel, 2010) and that this process is also limited by working memory capacity. Notably the exercise of model-based control is similar to existing definitions of self-regulation, as “the largely (but not exclusively) volitional act of managing attention and arousal in a manner that facilitates goal-directed behavior” (Berry et al., 2013, p. 2). An advantage of the reinforcement learning approach is its ability to formalize such behavior within a normative computational framework. It is important to reiterate here that, whilst we view model-based valuations as supporting future-oriented choice, we do not identify the *model-free* controller with an “impulsive system.” In our view both controllers share the same fundamental goals and it is the relative inflexibility of model-free decision-making which gives its responses their short-sighted character (Dayan et al., 2006).

### Novel behavioral predictors and interventions

Several additional approaches may yield novel behavioral markers of unhealthy choice. Unlike the *naïve* decision maker described above, people often demonstrate that they can predict their future tendencies, termed *sophistication*, for instance by choosing paths that remove their opportunity to make myopic choices, an activity referred to as *pre-commitment* (Ainslie, 2001; Ariely and Wertenbroch, 2002; Prelec and Bodner, 2003). For instance, a person attempting to abstain from smoking might throw away their cigarette packets. Pre-commitment would be expected to obscure real-world relationships between discount rates and myopic behavior, since at least a subset of sophisticated steep discounters would exhibit far–sighted real–world choices. Furthermore, within the model-based versus model-free framework above, we propose that the ability to predict and therefore pre-empt the influence of state changes on one's behavior is a key substrate of self-control. Economic theories of pre-commitment, often based on quasi-hyperbolic discounting, provide a useful conceptual framework for predicting the effects of varying degrees of sophistication on behavior (O'Donoghue and Rabin, 2003), and computational models of these processes (Kurth-Nelson and Redish, 2010) offer the potential to enrich predictions of health behavior.

An additional important influence not considered above is the effect of *internal* motivational states. The effects of motivational state on habitual responding are complex, having immediate effects on the vigor of responding, while having effects on choice by altering the utility of outcomes (see Niv et al., 2007). Models which formalize these effects remain the subject of ongoing theoretical work, though may eventually provide a valuable substrate for applied health behavior research.

Although not discussed in detail here, since many health-promoting behaviors are to a degree aversive, measures of dread (Berns et al., 2006; Story et al., 2013) might form a predictor of engagement in such behaviors. A complexity tending to preclude clear *a priori* predictions in this area is that, if dreading aversive health-promoting behaviors were to promote their avoidance (e.g., Kleinknecht, 1978), then dreading *illness* would be expected to have the opposite effect, promoting engagement in such behaviors. Perhaps reflecting these competing influences, Chapman and Coups (1999) report that rates of vaccination uptake were not significantly higher in individuals with negative time preference for illness, as compared to individuals with positive time preference for illness.

Finally, the account above focuses on instrumental learning. However, there is also evidence that animals use state-state, as well as state-action-state, associations to guide action. This third mode of learning, embodied by classical conditioning, is referred to as Pavlovian learning (Domjan, 2003). Based on state-state predictions, the Pavlovian controller initiates stereotyped actions directed toward obtaining predicted rewards. Crucially, unlike instrumental control, Pavlovian actions are initiated regardless of whether or not they lead to reward (Williams and Williams, 1969). The precise contributions of instrumental and Pavlovian effects to real-world choices are difficult to distinguish. Nevertheless the mechanism of choice inconsistency proposed above for the case of model-based and model-free interactions would remain largely equivalent for the case of interactions between model-based and Pavlovian decision-making (Dayan et al., 2006). An advantage of the reinforcement learning approach is its ability to generate simulations of these interactions over the course of learning and such models may yield parameters capable of explaining further variance in health behavior.

Novel interventions might be directed at specific constructs within the above framework, and indeed several existing health behavior interventions can be viewed in this manner. For example strategies aimed at making *healthy* choices habitual are already known to be effective (Lally et al., 2007). There is an urgent requirement for novel interventions capable of reducing goal-incongruent unhealthy choice, since the increasing burden of disease attributable to unhealthy behavior is placing unsustainable demands on existing healthcare systems (Smith et al., 2012). We propose that the identification of health decision-making phenotypes will play an important role in evaluating and optimizing the necessary interventions.

## Author contributions

All authors (Giles W. Story, Ivo Vlaev, Ben Seymour, Raymond J. Dolan, and Ara Darzi) contributed to the conception of the work. GS performed systematic review and drafted the work. All authors (Giles W. Story, Ivo Vlaev, Ben Seymour, Raymond J. Dolan, and Ara Darzi) were involved in revising the work critically for important intellectual content. All authors (Giles W. Story, Ivo Vlaev, Ben Seymour, Raymond J. Dolan, and Ara Darzi) gave final approval to the version of the manuscript being published and agree to be accountable for all aspects of the work.

### Conflict of interest statement

The authors declare that the research was conducted in the absence of any commercial or financial relationships that could be construed as a potential conflict of interest.
