# Supplementary material for: Does temporal discounting explain unhealthy behavior? A systematic review and reinforcement learning perspective
Source: Front Behav Neurosci. 2014 Mar 12;8:76. doi: 10.3389/fnbeh.2014.00076 (PMC3950931; doi:10.3389/fnbeh.2014.00076)
Supplement: Supplementary file 1 [file DataSheet1.DOCX]

**Supplementary Table 1**

*Studies Comparing Discount Rates and Smoking Behavior (N = 39)^*^*

| Study | Health outcome | Modality | Real/  Hypothetical | N | DF |
| --- | --- | --- | --- | --- | --- |
| Mitchell (1999) | Smokers vs non smokers | Money | Real (IC) | 40 | HYP, IP |
| Bickel, Odum & Madden (1999) | Current smokers vs ex-smokers vs never smokers | Money, cigarettes | Hypothetical | 66 | HYP |
| Odum, Madden & Bickel (2002) | Current smokers vs ex-smokers vs never smokers | Health | Hypothetical | 66 | EXP, HYP |
| Reynolds et al (2003) | Never smokers vs “triers” vs current smokers | Money | IC | 55 | HYP |
| Epstein et al (2003) | Smokers by amount smoked | Money | Hypothetical | 78 | HYP |
| Baker, Johnson & Bickel (2003) | Smokers vs never smokers | Health, money, cigarettes | Both | 60 | HYP |
| Mitchell (2004) | Nicotine deprivation in smokers | Money, cigarettes | Real (IC) | 11 | HYP, IP |
| Reynolds et al (2004) | Smokers vs never smokers | Money | Real | 54 | HYP |
| Reynolds (2004) | Adolescent and young adult smokers vs young adult non- smokers | Money | Hypothetical | 73 | HYP |
| Ohmura, Takahashi, Kitamura (2005) | Smokers vs never smokers; amount smoked | Money | Hypothetical | 50 | AUC,EXP, HYP |
| Reynolds (2006) | Smokers vs non smokers | Money | Real (EDT) | 30 | HYP |
| Heyman & Gibb (2006) | Smokers stratified by amount smoked | Money | Both | 71 | HYP |
| Field et al (2006) | Smokers vs abstinent smokers | Money, cigarettes | Hypothetical | 30 | AUC |
| Johnson, Bickel & Baker (2007) | Smokers vs non smokers; amount smoked | Money | Hypothetical | 90 | HYP |
| Reynolds et al (2007) | Smokers vs non smokers (adolescents) | Money | Hypothetical | 51 | AUC |
| Khwaja, Silverman & Sloan (2007) | Smokers vs ex-smokers vs never smokers | Money, health | Hypothetical | 431 | EXP |
| Yoon et al (2007) | Pregnant women and postpartum smoking relapse | Money | Hypothetical | 48 | HYP |
| Dallery & Raiff (2007) | Laboratory model of smoking abstinence reinforcement | Money | Hypothetical | 30 | HYP,AUC |
| Krishnan-Sarin et al (2007) | Smoking cessation in adolescents | Money | Both (EDT) | 30 | HYP |
| Reynolds et al (2008) | Smokers vs non smokers;children of smokers vs non smokers | Money | Hypothetical | 60 | HYP |
| Sweitzer et al (2008) | Never smokers vs “tried it” smokers vs regular smokers | Money | Hypothetical | 710 | HYP |
| Yi et al (2008) | Effects of smoking cessation on discounting | Money, cigarettes | Hypothetical | 56 | HYP |
| Mueller et al (2009) | Smoking abstinence in the laboratory | Money, cigarettes | Hypothetical | 19 | HYP |
| Mackillop & Kahler (2009) | Smokers (also drinking heavily) in smoking cessation programme | Money | Hypothetical | 57 | MCQ score |
| Jones et al (2009) | Smokers vs non smokers | Money | Hypothetical | 227 | HYP |
| Fields et al (2009) | Smokers vs non smokers (adolescents) | Money | EDT | 100 | AUC |
| Audrain-McGovern et al (2009) | Prospective study of smoking adoption (adolescents) | Money | Hypothetical | 947 | HYP |
| Reynolds et al (2009) | Smokers vs non smokers (mothers) | Money | Hypothetical | 30 | HYP |
| Rezfarnard et al (2010) | Heavily dependent vs lightly dependent smokers vs non-smokers | Money | Hypothetical | 89 | HYP |
| Stillwell & Turney (2012) | Smokers vs non smokers | Money | Hypothetical | 9454 | HYP, EXP |
| Yi & Landes (2012) | Smokers vs smoking abstinence | Money, cigarettes | Both | 28 | EXP |
| Wing et al (2012) | Smokers vs ex-smokers vs non smokers; schizophrenia vs controls | Money | Hypothetical | 130 | HYP |
| Reynolds & Fields (2012) | Adolescents experimenting with smoking | Money | Real (IC) | 141 | AUC |
| Moallem & Ray (2012) | Heavy drinkers, smokers and heavy drinking smokers | Money | Hypothetical | 387 | HYP |
| Sheffer et al (2012) | Cognitive behavioral abstinence program in smokers | Money | Both | 97 | HYP |
| Kang & Ikeda (2013) | Smoking frequency | Money | Hypothetical | 5670 | EXP^**^ |
| Kobiella et al (2013) | Smokers vs non-smokers (plus nicotine administration) | Money | Real (IC) | 76 | HYP |
| Brown & Adams (2013) | Prospective study of smoking cessation | Money | Other^***^ | 1817 | Other^**^ |

*Note* Studies are listed in chronological order. Abbreviations: EXP = Exponential function; HYP = Hyperbolic function; AUC = Area under curve; DF = Discount function; IP = indifference point; MCQ = Monetary Choice Questionnaire, a discounting method (Kirby et al., 1999); IC = Incentive compatible (a design in which a proportion of choices have real outcomes); EDT = Experiental Discounting Task (a task with real monetary rewards); * Note some studies appear in more than one supplementary table. **This study used a marker of hyperbolic discounting which denoted decreasing exponential discount rates. ***This study used the future time period deemed most important for financial planning as a proxy for time preference.

**Supplementary Table 2**

*Studies Comparing Discount Rates and Alcohol Use (N=17)*

| Study | Health outcome | Modality | Real/  hypothetical | N | DF |
| --- | --- | --- | --- | --- | --- |
| Vuchinich & Simpson (1998) | Light social drinkers vs heavy social drinkers vs problem drinkers | Money | Hypothetical | 48 | HYP |
| Petry (2001) | Alcoholics vs abstinent alcoholics vs controls | Money, alcohol | Hypothetical | 46 | HYP |
| Bjork et al (2004) | Abstinent alcohol dependent patients vs controls | Money | IC | 160 | HYP |
| Dom et al (2006) | Early vs late onset alcoholics vs controls | Money | Hypothetical | 142 | HYP |
| Mitchell et al (2005) | Abstinent alcoholics vs controls | Money | Hypothetical | 31 | E:L |
| Boettiger et al (2007) | Alcoholics vs controls | Money | Hypothetical | 19 | E:L |
| Mackillop et al (2007) | Social drinking vs hazardous drinking (students) | Money | Hypothetical | 93 | HYP |
| Field et al (2007) | Light vs heavy drinkers (adolescents) | Money, alcohol | Hypothetical | 66 | AUC |
| Rossow (2008) | Frequency of alcohol use, adverse consequences (adolescents) | Money | Hypothetical | 17413 | EXP |
| Bobova et al (2009) | Alcohol dependence, +/- previous childhood conduct disorder vs controls (young adults) | Money | Hypothetical | 426 | HYP |
| Mackillop et al (2010) | Heavy drinkers | Money | Hypothetical | 61 | MCQ |
| Moore & Cusens (2010) | Blood alcohol levels (social drinkers) | Money | Hypothetical | 46 | HYP |
| Fernie et al (2010) | Measures of alcohol use (social drinkers ) | Money | Hypothetical | 75 | AUC |
| Dennhardt & Murphy (2011) | Frequency of alcohol use, adverse consequences (undergraduates) | Money | Hypothetical | 206 | HYP |
| Yankelevitz, Mitchell & Zhang (2011) | Alcohol consumption (college students) | Money, alcohol | Hypothetical | 65 | AUC |
| Moallem & Ray (2012) | Heavy drinkers, smokers and heavy drinking smokers | Money | Hypothetical | 387 | HYP |
| Andrade et al. (2013) | History of alcohol problems or smoking in problem gamblers | Money | Hypothetical | 333 | HYP |

*Note* Studies are listed in chronological order. Abbreviations: EXP = Exponential function; HYP = Hyperbolic function; AUC = Area under curve; DF = Discount function; MCQ = Monetary Choice Questionnaire, a discounting method (Kirby et al., 1999); IC = Incentive compatible (a design in which a proportion of choices have real outcomes); E:L = early:late choice ratio

**Supplementary Table 3**

*Studies Comparing Discount Rates and Illicit Substance Misuse (N = 27)*

| Study | Health outcome | Modality | Real/  hypothetical | N | DF |
| --- | --- | --- | --- | --- | --- |
| Madden et al (1997) | Opiate dependent patients vs controls | Money, heroin | Hypothetical | 56 | HYP |
| Kirby, Petry & Bickel (1999) | Heroin users vs controls | Money | Real (IC) | 116 | HYP |
| Bretteville-Jensen (1999) | Active heroin users vs former heroin users vs controls | Money | Hypothetical | 270 | EXP |
| Petry & Casarella (1999) | Substance abusing gamblers vs substance abusing non gamblers vs controls | Money | Hypothetical | 81 | HYP |
| Petry (2001) | Gamblers with substance misuse vs gamblers without substance misuse | Money | Hypothetical | 50 | HYP |
| Giordano et al (2002) | Opiate deprivation in opioid dependent patients | Money, heroin | Hypothetical | 13 | HYP |
| Moeller et al (2002) | Cocaine dependent subjects vs controls | Money | Real | 74 | SCF |
| Petry (2003) | Current or former substance users vs controls | Health, money, freedom | Hypothetical | 141 | HYP |
| Kollins (2003) | Substance abuse (students) | Money | Hypothetical | 47 | HYP |
| Coffey et al (2003) | Cocaine users vs controls | Money, cocaine | Hypothetical | 25 | HYP |
| Kirby & Petry (2004) | Heroin & cocaine users vs alcoholics vs controls ; abstinence >14days | Money | Real (IC) | 145 | HYP |
| Bornovalova et al (2005) | Users of primary crack cocaine vs users of primary heroin | Money | Hypothetical | 27 | HYP |
| Heil et al (2006) | Cocaine dependent current users vs dependent abstinent for 30 days vs controls | Money | Hypothetical | 63 | HYP |
| Hoffman et al (2006) | Dependent metamphetamine users (2 week abstinent) vs controls | Money | Hypothetical | 82 | HYP |
| Monterosso et al (2007) | Methamphetamine users vs controls | Money | Hypothetical | 29 | HYP |
| Ledgerwood et al (2009) | Gamblers with substance misuse vs gamblers without substance misuse vs controls | Money | Hypothetical | 101 | AUC |
| Businelle et al (2010) | Smokers with SUD (including alcohol) vs smokers without SUD vs never smokers  with SUD vs controls (never smoked, no SUD) | Money | Hypothetical | 115 | HYP |
| Johnson et al (2010) | Current vs former marijuana users vs controls | Money, marijuana | Hypothetical | 88 | HYP |
| Washio et al (2011) | Prospective study of treatment for cocaine-dependent individuals | Money | Hypothetical | 36 | HYP |
| Stea, Hodgkins & Lambert (2011) | Cannabis, gambling and alcohol (students) | Money | Hypothetical | 218 | AUC |
| Camchong et al (2011) | Cocaine dependent individuals vs controls | Money | Not specified | 51 | HYP |
| Bickel et al (2011) | Working memory training in stimulant users | Money | Both | 27 | EXP-P |
| Johnson (2012) | Cocaine dependent individuals vs controls | Money | Both  (QODT, EDT) | 40 | HYP |
| Stanger et al (2012) | Prospective study of treatment for marijuana abuse in adolescents | Money, marijuana | Hypothetical | 165 | HYP |
| Landes, Christensen & Bickel (2012) | Prospective study of treatment in opioid dependent individuals | Money | Hypothetical | 159 | HYP |
| Peters et al (2013) | Prospective study of treatment for marijuana abuse in adults | Money | Real (EDT) | 93/61^*^ | HYP |
| Heinz et al (2013) | Prospective study of treatment for marijuana abuse in military veterans | Money | Hypothetical | 72 | HYP |

*Note* Studies are listed in chronological order. Abbreviations: SUD = substance use disorder; EXP = Exponential function; HYP = Hyperbolic function; AUC = Area under curve); IC = Incentive compatible (a design in which a proportion of choices have real outcomes); QODT = Quick Discounting Operant Task (Johnson, 2012); EDT = Experiential Discounting Task (a task with real monetary rewards); SCF = sooner choice frequency; EXP-P = Exponential power function. **N* = 93 pre-treatment, 61 post-treatment.

**Supplementary Table 4**

*Studies Comparing Discount Rates and Obesity or Eating Behavior (N = 12)*

| Study | Health outcome | Modality | Real/  hypothetical | N | DF |
| --- | --- | --- | --- | --- | --- |
| Bonato & Boland (1983) | Obese vs healthy weight children | Food/non-food | Real | 40 | SCF |
| Johnson, Parry & Drabman (1978) | Obese vs healthy weight children | Food/noon-food | Real | 142 | SCF |
| Epstein et al (2003) | BMI in smokers | Money | Hypothetical | 78 | HYP |
| Borghans & Golsteyn (2005) | BMI | Money | Hypothetical | 2059 | SCF |
| Nederkoorn et al (2006) | Obese vs healthy weight women | Money | Hypothetical | 51 | HYP |
| Weller et al (2008) | Obese vs healthy weight | Money | Hypothetical | 112 | AUC |
| Ikeda, Kang & Ohtake (2010) | BMI | Money | Hypothetical | 2987 | EXP |
| Rollins et al (2010) | Healthy weight women, *ad libitum* eating | Money | Hypothetical | 24 | HYP |
| Davis et al (2010) | Obese women +/- binge eating disorder vs healthy weight women | Money | Hypothetical | 209 | IP |
| Fields et al (2011) | Obese vs healthy weight adolescent smokers | Money | Real (IC) | 35 | AUC |
| Appelhans (2011) | Food reward sensitivity in obese women | Money | Hypothetical | 62 | AUC |
| Kulendran et al (2013) | Obese vs healthy weight adolescents | Money | Real | 53 | HYP |

*Note* Studies are listed in chronological order. Abbreviations: EXP = Exponential function; HYP = Hyperbolic function; AUC = Area under curve;

SCF = sooner choice frequency; IP = indifference point; IC = Incentive compatible; BMI = Body Mass Index

**Supplementary Table 5**

*Studies Comparing Discount Rates and Self-reported health, Preventative Health Behaviors or Multiple Behaviors (N =17)*

| Study | Health outcome | Modality | Real/  Hypothetical | *N* | DF |
| --- | --- | --- | --- | --- | --- |
| Fuchs (1982) | Self-rated health, smoking, dentist attendance, seatbelt wearing | Money | Hypothetical | 500 | EXP |
| Chapman & Coups (1999) | Influenza vaccination uptake | Monetary loss, flu illness | Hypothetical | 412 | EXP |
| Odum et al (2000) | Needle-sharing amongst heroin users | Money, heroin | Hypothetical | 32 | HYP |
| Chapman et al (2001) (1) | Influenza vaccination uptake | Monetary loss, flu illness | Hypothetical | 679 | IP |
| Chapman et al (2001) (2) | Hypertension medication adherence | Money, chest pain | Hypothetical | 195 | IP |
| Chapman et al (2001) (3) | Cholesterol medication adherence | Money, chest pain | Hypothetical | 137 | IP |
| Chesson et al (2006) | Risky sexual behaviors in teenagers | Money | Hypothetical | 1042 | EXP |
| Chao et al (2009) | Self-assessed morbidity and subjective survival probability | Money | Hypothetical | 175 | HYP |
| Axon et al (2009) | Hypertensive adults: BP checks, diet and exercise, compliance with doctors’ plans | Money | Hypothetical | 422 | EXP |
| Reimers et al (2009) | Age at first sexual activity, smoking, BMI, substance misuse | Money | Hypothetical | 42863 | SCF |
| Bradford (2010) | Cancer and cholesterol screening, flu vaccinations, dental visits, non-smoking , exercise | Money | Hypothetical | 987 | Utility |
| Daugherty & Brase (2010) | Tobacco, alcohol, drug use, exercise, breakfast, seatbelt, estimated longevity,  health concerns, sociosexual orientation | Money | Hypothetical | 467 | MCQ |
| Huckans et al (2010) | Hepatitis C status and substance misuse | Money | Hypothetical | 83 | HYP |
| Van der Pol (2011) | Self-assessed health | Money | Hypothetical | 2300 | EXP |
| Dierst-Davis et al (2011) | Homeless, substance-dependent men who have sex with men vs non-substance  dependent men with stable housing | Money | Hypothetical | 40 | HYP |
| Moallem & Ray (2012) | Heavy drinkers, smokers and heavy drinking smokers | Money | Hypothetical | 387 | HYP |
| Melanko & Larkin (2012) | Healthy lifestyle questionnaire | Money | Both (EDT) | 72 | AUC |

*Note* Studies are listed in chronological order. Abbreviations: EXP = Exponential function; HYP = Hyperbolic function; AUC = Area under curve);

IC = Incentive compatible (a design in which a proportion of choices have real outcomes); EDT = Experiental Discounting Task (a task with real monetary rewards).

**Estimated Future Illness Cost**

**Estimated Immediate Cost**
